# Effect of School-Based Education Intervention on Knowledge and Attitude Regarding Seasonal Influenza Vaccine Uptake Among Secondary Schoolgirl Students in Al-Ahsa, Saudi Arabia: A Quasi-experimental Study

**DOI:** 10.7759/cureus.68283

**Published:** 2024-08-31

**Authors:** Israa Al Ibrahim, Ahmed Z Al Saif, Rahma AlGadeeb, Abdulkareem J Al-Quwaidhi

**Affiliations:** 1 Preventive Medicine, Al-Ahsa Health Cluster, Al-Ahsa, SAU; 2 Population Health Management, Eastern Health Cluster, Dammam, SAU

**Keywords:** practice, influenza vaccination, knowledge, education, attitude

## Abstract

Background

School-based educational interventions are critical because they provide an opportunity to strengthen preventive measures by educating students about the importance of vaccination and promoting healthy practices within the community.

Aim

The study aimed to assess the effectiveness of influenza vaccination education in terms of knowledge and attitudes among secondary schoolgirl students in Al-Ahsa, Saudi Arabia.

Methods

This open-label, parallel-group, quasi-experimental study included 419 secondary school girls in Al-Ahsa, Saudi Arabia. The control group comprised 199 participants, while the intervention group comprised 220 participants. Both groups were administered a self-administered Arabic questionnaire prior to the study to collect information on participants' demographics, knowledge, attitudes, and practices regarding seasonal influenza and its vaccine. Subsequently, the intervention group was presented with a brief educational video and evaluated via a post-test. The primary outcomes were the students' knowledge and attitudes about seasonal influenza vaccines. The secondary outcomes were the participants' practices and reasons for not receiving the vaccine for seasonal influenza.

Results

Following an educational intervention about seasonal influenza and its vaccine, there was a statistical increase in knowledge and attitudes among students compared to a pre-intervention baseline. However, in both intervention and control groups, only a small proportion of participants had received the influenza vaccine, either once or on more than one occasion. Most participants employed additional preventive measures beyond vaccines; however, the majority also believed that vaccines were ineffective or perceived influenza as a relatively minor illness.

Conclusion

Implementing an influenza vaccination education program effectively enhances the knowledge and attitudes of secondary school female students in Al-Ahsa, Saudi Arabia. Nevertheless, further measures need to be taken to enhance the low vaccination uptake among the target population.

## Introduction

According to the WHO, influenza is a seasonal flu that affects people of all ages, usually 5-10% of adults and kids. It is responsible for a lot of seasonal flu epidemics around the world every year [1]. The Spanish flu pandemic of 1918-1919 was one of the most devastating and tragic pandemics in history, affecting an estimated three-quarters of the world's population [2]. The Centers for Disease Control and Prevention has documented additional instances of influenza in recent years, with the most recent pandemic occurring in 2009 and caused by the influenza A virus (H1N1, pdm09) [3].

Influenza infections across the world affect an average of five million people, with close to 500,000 deaths globally each year, most of which are children (65 years of age) [1]. Due to hospital admissions and productivity loss, influenza is responsible for enormous economic costs [4]. Influenza is a short-term illness that affects the upper respiratory system, resulting in inflammation as the body works to deliver immune cells to the infection site rapidly [5]. The immune system reacts, releasing cytokines and chemokines (interferon), which cause symptoms such as high fever, coryza, and body aches [6].

Influenza A, the most prevalent influenza virus in humans, is genetically unstable, and its mutation rate is over 300 times higher than that of influenza B. This is due to changes in its primary functional and antigenic proteins, which occur by two mechanisms: antigenic drift and antigenic shift [7]. Therefore, influenza vaccines are recommended by most healthcare providers and organizations so that people can be protected against this infection medically, economically, and socially. Vaccines work by ensuring the body develops antibodies about two weeks after administration, which protect against infection [8].

Vaccination is the most effective method of preventing influenza; its development has evolved over the years due to mutations in virus structures and new emerging strains [9]. The influenza vaccine aims to protect against disease; recent research advances have focused on creating a universal vaccine that offers protection against all influenza virus strains, addressing the issue of antigenic drift and shifts [6].

School-based educational interventions related to influenza are critical to reducing the spread of the virus and protecting the health of students, staff, and the broader community. Because influenza is highly contagious and can affect a significant portion of the student population, schools can serve as transmission hubs. Educating students about proper hygiene and the importance of vaccination can significantly reduce transmission rates. Additionally, informed students are more likely to encourage healthy practices in their families, positively impacting influenza prevention throughout the community [10-12].

Many regions worldwide experience influenza; the Middle East is no exception. Influenza remains an extreme threat in Saudi Arabia, which was one of the most affected countries during the 2009 epidemic. Almost 100 cases were reported in 2010, with 124 deaths [13]. To date, several other cases of influenza have been reported in Saudi Arabia, particularly in major cities and provinces across the country. This situation is exacerbated due to the massive yearly congregations of Muslims in the holy cities of Makkah and Madinah for Omera and Haj, when influenza strains may potentially be transported to the country [14]. Moreover, seasonal influenza at schools is a problematic issue, as close contact among scholars leads to the fast spread of the viral infection between them and later to the public [15].

In Saudi Arabia, there is a paucity of research on school-based interventions to promote influenza vaccination. Previous studies have not comprehensively evaluated the impact of educational interventions on vaccination uptake among students. This study aims to fill this gap by focusing on school-based educational interventions and their effectiveness in promoting seasonal influenza vaccination.

Incorporating vaccination interventions into school settings offers a valuable strategy for promoting public health, improving immunization rates, and reducing the burden of preventable diseases. By leveraging the accessibility and influence of schools, we can effectively protect children and adolescents while contributing to broader population health goals.

This study specifically targets secondary school girls in Al-Ahsa, Saudi Arabia. Females often play a key role in promoting health practices within their families and communities [16]. By educating and empowering female students, we can potentially amplify the impact of the intervention through their influence on family health behaviors.

This study aimed to evaluate the effectiveness of a school-based educational intervention about the seasonal influenza vaccine on knowledge and attitude among secondary school girls in Al-Ahsa, Saudi Arabia.

## Materials and methods

Ethics statement

The study was approved by the Institutional Review Board of King Fahad Hospital - Al Hofuf (31-EP-2024). In addition, we obtained an agreement from the General Administration of Education in Al-Ahsa Governorate. Before participation, assent from the student and informed consent from a parent/guardian were obtained. The participant information was anonymized and kept confidential.

Study design, setting, and date

This open-label, parallel-group, quasi-experimental study was conducted between December 2023 and January 2024 among secondary schoolgirls in Al-Ahsa, Saudi Arabia.

Sample size

The sample size for our study was calculated based on a medium effect size (Cohen's d = 0.5), which is commonly used in educational and behavioral research. With a significance level (α) of 0.05 and power (1-β) of 95%, the sample size required to detect a medium effect size was determined to be approximately 105 participants per group, with a total of 210 participants. However, to ensure that our study had robust power and could account for any potential variability or confounding factors, we doubled the sample size. The aim behind such a large sample size was to provide increased statistical power to detect the expected effect of the educational intervention.

Eligibility criteria

The study included 419 secondary schoolgirls (aged 14-19 years) in participating schools in first, second, and third grades. Students were eligible to participate if they were physically present in class during the intervention. We excluded students with documented mental or physical conditions that could impair their ability to understand the study and provide informed consent.

Sampling

We employed a multistage cluster sampling design to select schools in Al-Ahsa Governorate. The governorate is divided into four educational sectors: the middle, southern, northern, and eastern sectors. In the first stage, all schools within each sector were listed. In the second stage, a systematic sampling approach was used to select schools from each list. Finally, schools were randomly assigned to an intervention or a control group, with two schools allocated to each group.

Intervention

Google Forms platform was used to create the consent forms, and they were distributed electronically via WhatsApp to schoolgirls' parents through the designated school health counselor in each participating school.

A validated questionnaire in Arabic was adapted from a previous study [17]. We employed a pre-test/post-test design to evaluate the intervention's effectiveness. The allocation ratio was 1:1, with two schools assigned to the intervention group and two schools assigned to the control group. The questionnaire covered four major domains: demographics, knowledge, attitudes, and practices. Knowledge, attitudes, and practices domains have major questions, followed by minor questions. Participants’ answers in knowledge and attitude domains were multiple-choice questions, and the participants ticked for the appropriate answers.

After school visits were arranged, teachers prepared classrooms equipped with projectors for the intervention. The researchers began by introducing themselves and explaining the study procedure. Participants of the intervention group (n = 220) were then given a sheet of paper with pre-test questions, which they completed during the session. Then, a 4:40-minute Arabic video about influenza, developed by the Saudi MOH, was shown. It was followed by a question-and-answer session where students were allowed to ask questions so the researchers could clarify any doubts. Finally, participants of the intervention group completed the post-test questions. The control group subjects (n = 199) only received the pre-test to assess baseline knowledge without exposure to the intervention. The intervention was conducted over a single session.

For knowledge of seasonal influenza and influenza vaccination, each correct answer was scored as one, while incorrect answers were scored as zero. A total score of 65% and above was classified as “good”, while a score below 65% was classified as “poor”. Attitudes were classified as "positive" if there were four or more positive responses or "negative" if there were four or more negative responses to the seven attitude questions.

Outcomes

The primary outcomes were the secondary school students' knowledge and attitudes about seasonal influenza vaccines. The secondary outcomes were the participants' practices and reasons for not receiving the vaccine for seasonal influenzas.

Data analysis

The data were analyzed using the IBM SPSS Statistics (version 21). All statistical methods were two-tailed with an alpha level of 0.05, and significance was considered when the p-value was less than or equal to 0.05. Descriptive analysis was done by prescribing frequency distribution and percentage for personal and academic data. The Pearson chi-square and exact probability tests were used to compare groups, while the Mc-Nemar test was used to compare changes before and after intervention.

## Results

**Figure 1:**
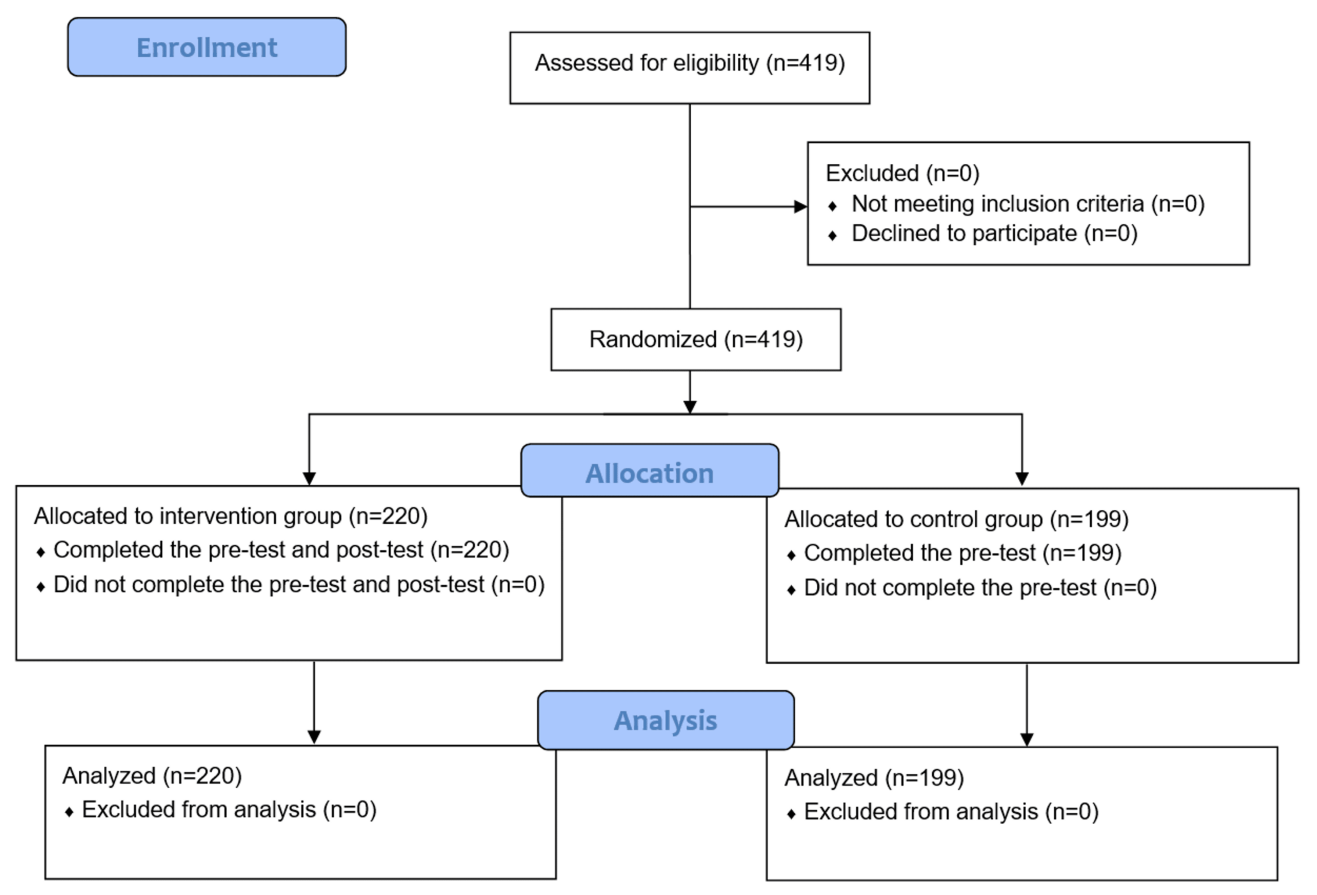
Flow diagram of the study participants.

A total of 419 secondary schoolgirls were allocated into two groups. The intervention group included 220 participants, while the control group included 199 participants. All participants were included in the final analysis (Figure 1).

The students’ ages ranged from 14 to 19 years, with a mean (SD) age of 16.1 (1.1) for the intervention group and 16.9 (1.1) for the control group (p=0.086). As for grade, 66.1% of the intervention group were in their first grade vs. 21.6% of the control group girls, and 23.9% of the intervention group were in their third grade compared to 65.8% of the control group girls (p=0.001, Table 1).

**Table 1:** Personal characteristics. * p<0.05 (significant); ^a ^Pearson’s chi-square test.

Personal data	Group				p-value^a^
	Control (n=199)		Intervention (n=220)		
	N	%	N	%	
Age in years					0.086
14-15	27	13.6%	67	30.5%	
16-17	103	51.8%	119	54.1%	
18-19	69	34.7%	34	15.5%	
Grade					0.001*
1^st^	43	21.6%	144	66.1%	
2^nd^	25	12.6%	22	10.1%	
3^rd^	131	65.8%	52	23.9%	

Table 2 demonstrates knowledge about seasonal influenza between the intervention and the control group at baseline assessment. Compared to the intervention group, the control group had a significantly higher baseline knowledge regarding the virus as a cause of influenza (p=0.003), "Flu can spread from one person to another” (p=0.032), "Flu occurs at a certain period of the year (0.048)", and Headache, sore throat, and fatigue as symptoms (p=0.002, 0.001, and 0.036, respectively).

**Table 2:** Baseline knowledge about seasonal influenza. * p<0.05 is significant; ^a^ Pearson’s chi-square test; ^ Exact probability test

Seasonal influenza knowledge	Control (n=199)				Intervention (n=220)				
	Incorrect / Don’t know		Correct answer		Incorrect / Don’t know		Correct answer		p-value
	N	%	N	%	N	%	N	%	
General knowledge									
Flu is caused by a virus	14	7.0%	185	93.0%	35	15.9%	185	84.1%	0.003*^a^
Flu can spread from one person to another	6	3.0%	193	97.0%	17	7.7%	203	92.3%	0.032*^a^
Flu can be prevented	64	32.2%	135	67.8%	75	34.1%	145	65.9%	0.557^a^
Flu is the same as a common cold	152	76.4%	47	23.6%	150	68.2%	70	31.8%	0.048*^a^
Flu occurs at a certain period of the year	28	14.1%	171	85.9%	50	22.7%	170	77.3%	0.023*^a^
Clinical presentation									
Headache	25	12.6%	174	87.4%	52	23.6%	168	76.4%	0.002*^a^
Running nose	9	4.5%	190	95.5%	16	7.3%	204	92.7%	0.221^
Sneezing	28	14.1%	171	85.9%	33	15.0%	187	85.0%	0.652^a^
Sore throat	22	11.1%	177	88.9%	49	22.3%	171	77.7%	0.001*^a^
Muscle ache	83	41.7%	116	58.3%	106	48.2%	114	51.8%	0.107^a^
Fever	187	94.0%	12	6.0%	208	94.5%	12	5.5%	0.741^a^
Fatigue	46	23.1%	153	76.9%	70	31.8%	150	68.2%	0.036*^a^

Table 3 shows comparable knowledge of the seasonal influenza vaccine between the intervention and the control groups. The control group significantly thought that there were side effects from influenza vaccination than the intervention group (80.4% vs. 67.7%, p=0.003).

**Table 3:** Baseline knowledge about the seasonal influenza vaccine. * p<0.05 is significant; ^a^ Pearson’s chi-square test.

Seasonal influenza vaccine	Control				Intervention				
	Incorrect / Don’t know		Correct answer		Incorrect / Don’t know		Correct answer		p-value^a^
	N	%	N	%	N	%	N	%	
General knowledge									
Is the flu vaccine safe?	95	47.7%	104	52.3%	109	49.5%	111	50.5%	0.712
Does annual influenza vaccination prevent infection with seasonal influenza?	134	67.3%	65	32.7%	133	60.5%	87	39.5%	0.143
Do you think that influenza vaccination prevents the risks of contracting seasonal influenza?	89	44.7%	110	55.3%	104	47.3%	116	52.7%	0.601
Do you think there are side effects from influenza vaccination?	39	19.6%	160	80.4%	71	32.3%	149	67.7%	0.003*
Soreness/swelling at the injection site	92	46.2%	107	53.8%	113	51.4%	107	48.6%	0.294
Side effects									
Fever	62	31.2%	137	68.8%	66	30.0%	154	70.0%	0.798
Headache	136	68.3%	63	31.7%	152	69.1%	68	30.9%	0.869
Nausea	114	57.3%	85	42.7%	140	63.6%	80	36.4%	0.184
Muscle ache	99	49.7%	100	50.3%	121	55.0%	99	45.0%	0.282
Do you expect other side effects?	141	70.9%	58	29.1%	150	68.2%	70	31.8%	0.553
For how long the vaccine can protect?	70	35.2%	129	64.8%	76	34.5%	144	65.5%	0.892

Table 4 shows that the intervention group had significantly less overall knowledge about seasonal influenza than the control group (70.9% vs. 82.9%, p=0.004). No significant difference regarding Influenza vaccine immunization knowledge was reported (p=0.602).

**Table 4:** Overall baseline knowledge level about seasonal influenza and its vaccine. * p<0.05 is significant; ^a^ Pearson’s chi-square test.

Knowledge domain	Control		Intervention		p-value^a^
	N	%	N	%	
Seasonal influenza knowledge level					0.004*
Poor	34	17.1%	64	29.1%	
Good	165	82.9%	156	70.9%	
Influenza vaccine immunization knowledge level					0.602
Poor	142	71.4%	162	73.6%	
Good	57	28.6%	58	26.4%	

After the intervention, the intervention group had statistically higher knowledge about seasonal influenza, including "Flu can be prevented", "Flu is the same as a common cold", and its clinical presentation, comprising headache, muscle aches, and fever, compared to before the intervention (p<0.05, Table 5).

**Table 5:** Knowledge about seasonal influenza before and after intervention (n=220). * p<0.05 is significant; ^a^ Mc-Nemar test.

Seasonal influenza knowledge	Pre-intervention				Post-intervention				
	Incorrect / Don’t know		Correct answer		Incorrect / Don't know		Correct answer		p-value^a^
	N	%	N	%	N	%	N	%	
General knowledge									
Flu is caused by a virus	35	15.9%	185	84.1%	33	15.0%	187	85.0%	0.884
Flu can spread from one person to another	17	7.7%	203	92.3%	46	20.9%	174	79.1%	0.001*
Flu can be prevented	75	34.1%	145	65.9%	61	27.7%	159	72.3%	0.003*
Flu is the same as a common cold	150	68.2%	70	31.8%	143	65.0%	77	35.0%	0.047*
Clinical presentation									
Headache	52	23.6%	168	76.4%	27	12.3%	193	87.7%	0.046*
Running nose	16	7.3%	204	92.7%	36	16.4%	184	83.6%	0.044*
Sneezing	33	15.0%	187	85.0%	41	18.6%	179	81.4%	0.096
Sore throat	49	22.3%	171	77.7%	55	25.0%	165	75.0%	0.063
Muscle ache	106	48.2%	114	51.8%	83	37.7%	137	62.3%	0.039*
Fever	208	94.5%	12	5.5%	189	85.9%	31	14.1%	0.041*
Fatigue	70	31.8%	150	68.2%	78	35.5%	142	64.5%	0.084

Table 6 illustrates a significant enhancement in understanding seasonal influenza vaccines following the intervention. This is evidenced by a marked increase in the number of students who correctly could answer the question, "Is the flu vaccine safe?". The participants were also asked whether the annual influenza vaccination prevents infection with seasonal influenza, whether they believe it prevents the risks of contracting it, and whether they had experienced soreness or swelling at the injection site. Additionally, there was a statistical increase in knowledge regarding the occurrence of nausea as a side effect, with a higher proportion of participants reporting this after the intervention (49.1% vs. 36.4%; p=0.002).

**Table 6:** Knowledge about seasonal influenza vaccine before and after intervention (n=220). * p<0.05 is significant; ^a^ Pearson’s chi-square test.

Seasonal influenza vaccine	Pre- intervention				Post-intervention				
	Incorrect / Don’t know		Correct answer		Incorrect / Don’t know		Correct answer		p-value^a^
	N	%	N	%	N	%	N	%	
General knowledge									
Is the flu vaccine safe?	84	38.2%	136	61.8%	109	49.5%	111	50.5%	0.001*
Does annual influenza vaccination prevent infection with seasonal influenza?	114	51.8%	106	48.2%	133	60.5%	87	39.5%	0.003*
Do you think that influenza vaccination prevents the risks of contracting seasonal influenza?	94	42.7%	126	57.3%	104	47.3%	116	52.7%	0.048*
Do you think there are side effects from influenza vaccination?	90	40.9%	130	59.1%	71	32.3%	149	67.7%	0.063
Soreness/swelling at the injection site	97	44.1%	123	55.9%	113	51.4%	107	48.6%	0.039*
Side effects									
Fever	79	35.9%	141	64.1%	66	30.0%	154	70.0%	0.126
Headache	134	60.9%	86	39.1%	152	69.1%	68	30.9%	0.069
Nausea	112	50.9%	108	49.1%	140	63.6%	80	36.4%	0.002*
Muscle ache	110	50.0%	110	50.0%	121	55.0%	99	45.0%	0.087
Do you expect other side effects?	166	75.5%	54	24.5%	150	68.2%	70	31.8%	0.057
For how long the vaccine can protect?	92	41.8%	128	58.2%	76	34.5%	144	65.5%	0.528

After the intervention, there was a statistically higher positive attitude about the seasonal influenza vaccine among the study girls than before. Additionally, there was a marked increase in students who disagreed with the negative attitude about seasonal influenza vaccination (Table 7).

**Table 7:** Attitude about the seasonal influenza vaccine before and after intervention (n=220). * p<0.05 is significant; ^a^ Mc-Nemar test.

Attitude	Pre-intervention						Post-intervention						
	Disagree		Agree		Unsure		Disagree		Agree		Unsure		p-value^a^
	N	%	N	%	N	%	N	%	N	%	N	%	
Influenza vaccination is important and should be taken yearly	95	43.2%	54	24.5%	71	32.3%	79	35.9%	92	41.8%	49	22.3%	0.001*
Influenza vaccine prevents serious complications associated with seasonal influenza	49	22.3%	102	46.4%	69	31.4%	46	20.9%	115	52.3%	59	26.8%	0.437
Seasonal influenza vaccination causes side effects and effects, which is why I do not want to take the vaccine	48	21.8%	114	51.8%	58	26.4%	65	29.5%	90	40.9%	65	29.5%	0.048*
All people should receive influenza vaccine	98	44.5%	67	30.5%	55	25.0%	60	27.3%	86	39.1%	74	33.6%	0.001*
Flu is a mild illness and therefore vaccination is not necessary	104	47.3%	61	27.7%	55	25.0%	100	45.5%	58	26.4%	62	28.2%	0.627
I don’t need the flu vaccine because I have life immunity against the flu	71	32.3%	96	43.6%	53	24.1%	80	36.4%	69	31.4%	71	32.3%	0.019*
Do you intend to get vaccinated in the coming years?	108	49.1%	52	23.6%	60	27.3%	71	32.3%	64	29.1%	85	38.6%	0.001*

Table 8 shows that a low percentage of the intervention group received the vaccine once or more than once compared to the control group (16.1% and 25.2% vs. 23.1% and 19.1%, p=0.0186). As for reasons of not having the vaccine, the most reported among the two study groups were practicing other preventive methods instead of vaccination (51.6% and 58.5%, respectively), not necessary because flu is just a minor illness (39.1% and 45.3%), and thinking that the vaccine is not effective (37.5% and 47.2%) with no statistical significance (all p>0.05).

**Table 8:** Seasonal influenza vaccination practice and reasons for not receiving the vaccine. ^a^ Pearson’s chi-square test.

Practice	Group				p-value^a^
	Control (n=199)		Intervention (n=220)		
	N	%	N	%	
Have you received the seasonal influenza vaccine before?					0.186
No	53	26.6%	64	29.4%	
Once	46	23.1%	35	16.1%	
More than once	38	19.1%	55	25.2%	
Unsure	62	31.2%	64	29.4%	
Reasons for not receiving the vaccine					
It is not necessary because flu is just a minor illness	24	45.3%	25	39.1%	0.497
The vaccine is not effective	25	47.2%	24	37.5%	0.291
Fear of needles and injection	19	35.8%	13	20.3%	0.061
Because I practice other preventive methods instead of vaccination	31	58.5%	33	51.6%	0.454
Because whoever has previously been vaccinated with seasonal influenza is still in effect in the body, there is no need to revaccinate again.	13	24.5%	12	18.8%	0.448
Because I had taken the vaccination previously and it resulted in an adverse reaction after the vaccination on the first attempt	10	18.9%	10	15.6%	0.643

## Discussion

Influenza remains one of the top 10 global diseases. Vaccinating students reduces influenza transmission among the population. This study evaluated the effectiveness of a school-based education intervention about seasonal influenza vaccines on knowledge, attitude, and practice among secondary school girls in Al-Ahsa, Saudi Arabia.

Our main findings were that influenza vaccine education statistically improved the knowledge and attitude of secondary school girls in Al-Ahsa, Saudi Arabia. However, only a minority of participants reported receiving the influenza vaccine, either once or on more than one occasion. Most participants indicated that they had employed additional preventive measures other than vaccines. Additionally, a large proportion believed that vaccines were ineffective or considered influenza a trivial illness. The perception that vaccines are ineffective or that influenza is a trivial illness may reflect cultural factors. In Saudi Arabia, as in many other countries, cultural beliefs and misconceptions about vaccines can have a significant impact on vaccination rates. It has been shown that cultural norms, religious beliefs, and lack of awareness can contribute to vaccine hesitancy [18]. Several studies have highlighted the influence of cultural factors on vaccine uptake. Alamir [19] found that vaccine hesitancy in Saudi Arabia is often related to a belief that the potential adverse effects of vaccination outweigh the protective benefits against diseases.

The majority of students in both groups demonstrated a satisfactory understanding of the influenza virus yet exhibited limited knowledge regarding the influenza vaccine. Assaf et al. [20] attributed this to the fact that students acquired some of this knowledge during their biology courses at school. Similarly, Alhatim et al. [17] reported high levels of knowledge about the influenza virus, with nearly 90% of participants demonstrating an understanding that it causes influenza and over 96% aware of its high transmissibility among close contacts. However, only slightly more than half of the participants believed that the flu could be prevented, and nearly one-third mistakenly believed that the flu was similar to the common cold. Additionally, Aljamili [21] found that 86.9% of participants believed that influenza was a highly contagious disease that could potentially lead to hospitalization.

There is a notable discrepancy in knowledge about the influenza vaccine among different population groups. Alhatim et al. [17] found that 81.7% of their Saudi participants were aware of the availability of a flu vaccine. Of these, 71.5% believed the vaccine was safe, although slightly less than half thought it was useless. Alqahtani et al. [14] demonstrated that half of their population knew that the vaccine was the most effective method to prevent complications from influenza. Nevertheless, nearly half of the participants had not received the influenza vaccine.

Regarding the attitudes of students toward seasonal influenza prior to the intervention, Assaf et al. [20] discovered that 46.8% of school students expressed concern about contracting influenza, while 36.2% exhibited no such concern, indicating that their South Lebanon society underestimated the disease. Nevertheless, Ma et al. [22] revealed that over half of the young workers expressed concern about contracting influenza, while a minority exhibited a lack of concern in South China.

Education of secondary school girls effectively improved knowledge and attitudes regarding the influenza virus and its vaccine. Similarly, Assaf et al. [20] reported that students demonstrated enhanced knowledge and attitude regarding seasonal influenza viruses and the vaccine following the implementation of an educational intervention in Lebanon. This may be attributed to the students' greater interest in learning about the vaccine than in acquiring knowledge about the infection. The results demonstrated this approach's efficacy in improving the population's knowledge and attitude regarding influenza infection and vaccination. Similarly, Afonso et al. [23] revealed that implementing a multimodal intervention to educate and emphasize the significance of influenza vaccination to medical students at an early stage of their careers is an effective approach that is both straightforward and straightforward implement. In pregnant women, Wong et al. [24] reported that brief education effectively improved vaccination uptake. In a meta-analysis, Zhou et al. [25] demonstrated that educational interventions delivered via correspondence were effective. The combination of simple vaccine availability with educational messaging has the potential to enhance their effectiveness.

In the present study, the proportion of individuals who received the vaccine on a single occasion or one subsequent occasion was below 50%. In Riyadh, Alhatim et al. [17] reported that 43.3% of participants had received the influenza vaccine at some point. The prevalence rates reported by Alqahtani et al. [14], Sagor et al. [26], and Alljamili [21] were 44.53%, 36.7%, and 55%, respectively. In China, Ma et al. [22] found that the lack of awareness considerably impacted the voluntary vaccination rate. Similarly, Ermenlieva et al. [27] indicated that a lack of knowledge was associated with low vaccination rates, regardless of the predominantly positive beliefs and attitudes. Moreover, Sales et al. [28] discovered that individuals who held positive beliefs regarding the safety and efficacy of the influenza vaccine were more likely to receive the vaccine. The provision of public health education and information on vaccines, including recommendations for influenza vaccination in specific populations and the optimal time to receive the vaccine, also encouraged participants to get vaccinated. 

Various reasons were identified, with differences in perception observed across the nation. The reasons for the low uptake of vaccines were found to be the majority of our participants' use of additional preventive measures, the belief that vaccines were ineffective, and the perception of influenza as a relatively minor illness. Bukhsh et al. [29] reported that 35% of Pakistani parents did not consider vaccines essential for the well-being of their children. In Saudi Arabia, Alhatim et al. [17] found that 28.8% of the participants believed that the influenza vaccine was unnecessary to prevent influenza. Alabbad et al. [30] reported that the Saudi public rejected the influenza vaccine because of their perception that the vaccine did not provide any benefits, their belief in their good health, and their concerns about possible serious side effects associated with the vaccine. Meanwhile, Sales et al. [28] showed that Saudi individuals who believed that vaccination was safe, effective, and administered in a specific season and were aware of the need to be vaccinated were more likely to have received the influenza vaccine. Al Awaidy et al. [31] healthcare personnel encounter supplementary challenges, including a lack of conviction and logistical impediments such as inadequate equipment, time limitations, and infrastructure. In contrast, the primary challenge encountered by the public was a lack of awareness. Furthermore, these findings support the present study, indicating that any negative views or attitudes were associated with decreased vaccination rates. Riccò et al. [32] reported that over 67% of teachers indicated their vaccination motivation was to avoid infection. However, the study revealed a significant deficit in teachers' knowledge base regarding the mechanisms by which vaccines protect individuals against seasonal influenza. This lack of knowledge may have influenced their perceptions and attitudes toward vaccination, which could potentially be transmitted to their students.

A paucity of studies has evaluated secondary school students' knowledge and attitudes toward influenza vaccination. This research represents the first study of the efficacy of an educational intervention on knowledge, attitude, and practice related to seasonal influenza and vaccination among female secondary school students in Al-Ahsa, Saudi Arabia. Although the intervention effectively enhanced knowledge and attitudes in certain areas, the overall findings present a more complex picture. The present study offers compelling evidence that brief educational interventions can effectively enhance students' knowledge and attitudes regarding influenza vaccination rates. This underscores the necessity for more targeted strategies to address the misconceptions surrounding the vaccine's effectiveness and the importance of vaccination in preventing the spread of the virus.

Limitations

This study included a relatively small sample size and was conducted in a single Saudi region. The lack of blinding of participants and the absence of any educational intervention for the control group could introduce bias. Furthermore, the survey data were self-reported, which present a challenge in verifying the accuracy of students' information. The focus on adolescent girls may limit the generalizability of the findings to broader populations. It would be beneficial for future research to adopt a longitudinal approach to assess the long-term impact of educational interventions on vaccination uptake and explore strategies to enhance vaccine acceptance among diverse demographic groups.

## Conclusions

The educational intervention resulted in a significant improvement in the knowledge and attitudes of secondary schoolgirls in Al-Ahsa, Saudi Arabia, regarding seasonal influenza and its vaccine.

These findings emphasize the necessity of school-based educational programs to enhance awareness and attitudes regarding influenza vaccination. However, additional strategies are required to address the persistent barriers to vaccine uptake, such as misconceptions about vaccine efficacy and the perceived insignificance of influenza. It would be beneficial for future interventions to focus on reinforcing the importance of vaccination and addressing specific concerns in order to increase vaccination rates among adolescents.

## References

[1] Castro-Sánchez E, Chang PW, Vila-Candel R, Escobedo AA, Holmes AH (2016). Health literacy and infectious diseases: why does it matter?. Int J Infect Dis.

[2] Nasiri MJ, Danaei B, Deravi N, Chirani AS, Bonjar AH, Khoshgoftar Z, Karimi F (2022). Impact of educational interventions on the prevention of influenza: a systematic review. Front Public Health.

[3] Wang M, Han X, Fang H (2018). Impact of health education on knowledge and behaviors toward infectious diseases among students in Gansu Province, China. Biomed Res Int.

[4] Houser K, Subbarao K (2015). Influenza vaccines: challenges and solutions. Cell Host Microbe.

[5] Lafond KE, Nair H, Rasooly MH (2016). Global role and burden of influenza in pediatric respiratory hospitalizations, 1982-2012: a systematic analysis. PLoS Med.

[6] Baldo V, Bertoncello C, Cocchio S, Fonzo M, Pillon P, Buja A, Baldovin T (2016). The new pandemic influenza A/(H1N1)pdm09 virus: is it really "new"?. J Prev Med Hyg.

[7] Ruf BR, Szucs T (2009). Reducing the burden of influenza-associated complications with antiviral therapy. Infection.

[8] Morris DE, Cleary DW, Clarke SC (2017). Secondary bacterial infections associated with influenza pandemics. Front Microbiol.

[9] Boktor SW, Hafner JW (2023). Influenza. StatPearls.

[10] Drake JW (1993). Rates of spontaneous mutation among RNA viruses. Proc Natl Acad Sci U S A.

[11] Jefferson T, Rivetti A, Di Pietrantonj C, Demicheli V (2018). Vaccines for preventing influenza in healthy children. Cochrane Database Syst Rev.

[12] Duque J, McMorrow ML, Cohen AL (2014). Influenza vaccines and influenza antiviral drugs in Africa: are they available and do guidelines for their use exist?. BMC Public Health.

[13] AlMazroa MA, Memish ZA, AlWadey AM (2010). Pandemic influenza A (H1N1) in Saudi Arabia: description of the first one hundred cases. Ann Saudi Med.

[14] Alqahtani AS, Althobaity HM, Al Aboud D, Abdel-Moneim AS (2017). Knowledge and attitudes of Saudi populations regarding seasonal influenza vaccination. J Infect Public Health.

[15] Ide K, Yamada H, Matsushita K (2014). Effects of green tea gargling on the prevention of influenza infection in high school students: a randomized controlled study. PLoS One.

[16] Caldwell JC, Caldwell P (2024). Roles of women, families, and communities in preventing illness and providing health services in developing countries. The Epidemiological Transition: Policy and Planning Implications for Developing Countries: Workshop Proceedings.

[17] Alhatim N, Al-Bashaireh AM, Alqudah O (2022). Knowledge, attitude, and practice of seasonal influenza and influenza vaccine immunization among people visiting primary healthcare centers in Riyadh, Saudi Arabia. PLoS One.

[18] Galagali PM, Kinikar AA, Kumar VS (2022). Vaccine hesitancy: obstacles and challenges. Curr Pediatr Rep.

[19] Alamir AA (2024). Childhood vaccination hesitancy in Saudi Arabia: are we still facing a problem? A narrative review. Saudi Med J.

[20] Assaf I, Moussa S (2020). Effectiveness of a health education intervention about influenza and vaccine in secondary schools in South Lebanon. BAU J - Health Wellbeing.

[21] Aljamili AA (2020). Knowledge and practice toward seasonal influenza vaccine and its barriers at the community level in Riyadh, Saudi Arabia. J Family Med Prim Care.

[22] Ma Y, Li T, Chen W, Chen J, Li M, Yang Z (2018). Knowledge, attitudes and practices (KAP) toward seasonal influenza vaccine among young workers in South China. Hum Vaccin Immunother.

[23] Afonso N, Kavanagh M, Swanberg S (2014). Improvement in attitudes toward influenza vaccination in medical students following an integrated curricular intervention. Vaccine.

[24] Wong VW, Fong DY, Lok KY (2016). Brief education to promote maternal influenza vaccine uptake: a randomized controlled trial. Vaccine.

[25] Zhou X, Zhao X, Liu J, Yang W (2020). Effectiveness of educational intervention on influenza vaccine uptake: a meta-analysis of randomized controlled trials. Iran J Public Health.

[26] Sagor KH, AlAteeq MA (2018). Beliefs, attitudes, and barriers associated with the uptake of the seasonal influenza vaccine among patients visiting primary healthcare clinics. Saudi Med J.

[27] Ermenlieva NM, Tsankova GS, Todorova TT (2019). Seasonal influenza vaccination: knowledge, attitude and practice in Varna, Bulgaria. Ther Adv Vaccines Immunother.

[28] Sales IA, Syed W, Almutairi MF, Al Ruthia Y (2021). Public knowledge, attitudes, and practices toward seasonal influenza vaccine in Saudi Arabia: a cross-sectional study. Int J Environ Res Public Health.

[29] Bukhsh A, Rehman H, Mallhi TH (2018). Parents' attitude, awareness and behaviour towards influenza vaccination in Pakistan. Hum Vaccin Immunother.

[30] Alabbad AA, Alsaad AK, Al Shaalan MA, Alola S, Albanyan EA (2018). Prevalence of influenza vaccine hesitancy at a tertiary care hospital in Riyadh, Saudi Arabia. J Infect Public Health.

[31] Al Awaidy S, Althaqafi A, Dbaibo G (2018). A snapshot of influenza surveillance, vaccine recommendations, and vaccine access, drivers, and barriers in selected Middle Eastern and North African countries. Oman Med J.

[32] Riccò M, Vezzosi L, Gualerzi G, Signorelli C (2017). Knowledge, attitudes and practices (KAP) towards vaccinations in the school settings: an explorative survey. J Prev Med Hyg.

